# Hip Resurfacing Arthroplasty and Perioperative Blood Testing

**DOI:** 10.1155/2014/109378

**Published:** 2014-10-01

**Authors:** Andrew Cook, Steven Cook, Ian Smith, Patrick Weinrauch

**Affiliations:** ^1^Toowoomba Base Hospital, QLD, Australia; ^2^St. Andrew's War Memorial Hospital, 457 Wickham Terrace, Spring Hill, QLD, Australia; ^3^St. Andrew's Medical Institute, QLD, Australia; ^4^Brisbane Hip Clinic, QLD, Australia; ^5^School of Medicine, Griffith University, QLD, Australia

## Abstract

It is standard practice in many institutions to routinely perform preoperative and postoperative haemoglobin level testing in association with hip joint arthroplasty procedures. It is our observation, however, that blood transfusion after uncomplicated primary hip arthroplasty in healthy patients is uncommon and that the decision to proceed with blood transfusion is typically made on clinical grounds. We therefore question the necessity and clinical value of routine perioperative blood testing about the time of hip resurfacing arthroplasty. We present analysis of perioperative blood tests and transfusion rates in 107 patients undertaking unilateral hybrid hip resurfacing arthroplasty by the senior author at a single institution over a three-year period. We conclude that routine perioperative testing of haemoglobin levels for hip resurfacing arthroplasty procedures does not assist in clinical management. We recommend that postoperative blood testing only be considered should the patient demonstrate clinical signs of symptomatic anaemia or if particular clinical circumstances necessitate.

## 1. Introduction

Hip resurfacing arthroplasty, when indicated, is usually recommended in younger and healthier patient populations with less medical comorbidity. Hip resurfacing arthroplasty, in comparison to conventional total hip replacement, typically requires additional soft tissue releases and surgical dissection and therefore can be associated with increased blood loss [1–5].

In many institutions it is standard management in the perioperative period associated with major interventions such as hip joint arthroplasty to measure haemoglobin levels prior to and just after surgery. The rationale for such measurement is to assist in identification of patients with anaemia that may benefit from allogenic blood transfusion. There are, however, risks associated with allogenic blood transfusion and therefore a growing trend to rationalise the use of this limited resource [6–9].

Particularly in younger and healthier population groups, the numeric value or relative decrease in haemoglobin levels around the time of surgical intervention is often of less clinical consequence due to a greater physiologic capacity to manage with moderate volume blood loss. The decision to proceed with blood transfusion in these circumstances is more often guided by clinical observations of symptomatic anaemia. Particularly in younger patients without significant medical comorbidity, postoperative haemoglobin levels should be considered a relative guide to overall patient assessment and clinical management, rather than an absolute indicator to the requirement of blood transfusion.

It has been our observation for patients undertaking resurfacing arthroplasty of the hip that the degree of blood loss is typically modest and the requirement for blood transfusion is uncommon, as patients rarely develop clinical symptoms of anaemia. In this context we question the value or rationale of routine haemoglobin testing in the perioperative period associated with resurfacing arthroplasty.

## 2. Methods

A retrospective review of consecutive patients undertaking unilateral hybrid hip resurfacing arthroplasty under the care of the senior author (PW) over a three-year period from June 2009 to June 2012 was undertaken. Patients undergoing bilateral simultaneous hip resurfacing, simultaneous contralateral side procedures, or management with uncemented resurfacing or mid-head resecting implants were excluded. Only patients managed by the senior author at a single institution (St. Andrew's War Memorial Hospital, Brisbane) were included in the study.

All procedures were undertaken under a general anaesthetic with preoperative fascia lata block administered by the anaesthetist on induction. Posterior surgical approach was used in all patients. Typically the approach included partial recession of the femoral attachment of the gluteus maximus tendon from the femur and complete anterior capsulotomy. All patients entered into the study were managed with hybrid resurfacing arthroplasty consisting of a monoblock uncemented acetabular component and a cemented monoblock femoral component using Antibiotic Simplex. The femoral component was impacted at 90–120 seconds after the mixing of cement. No topical or systemic procoagulants, wound drainage catheters, or cell salvage methods were used in any patient. Infiltration of anaesthetic mixture into the wound was conducted by the Surgeon during the procedure (ropivacaine 0.2% 150 mls, ketorolac 30 mgs, morphine 5 mg, and adrenaline 1/1000 0.5 mls). General anaesthesia was undertaken using total intravenous methods with aggressive crystalloid volume loading (typically 3 litres of Hartmann's solution during surgery) to facilitate early mobilisation by correction of volume depletion as a result of fasting and intraoperative blood loss. Patients were usually mobilised within 4–6 hours after surgery. Chemical thromboprophylaxis included rivaroxaban 10 mg daily for 30 days, starting on the second postoperative day. All patients were managed with short leg graduated compression stockings for 6 weeks and pneumatic leg or foot intermittent compression devices while in hospital.

All patients underwent a blood test on admission prior to surgery and a second blood test taken in the morning of their first postoperative day. Recorded haemoglobin and albumin levels were collated onto a database for further analysis. Further outcome measures recorded included length of stay, transfusion rate, readmission rate within 90 days of intervention, joint infections, and arthroplasty revision recorded from the Australian National Joint Replacement Registry.

Criteria for discharge from hospital included adequate analgesia with demonstrated safe independent mobility using crutches and a suitable home environment with supervision.

Approval for the conduct of this research was obtained from the St. Andrew's Medical Research Institute Ethics Committee (Approval number 2012.20.59).

## 3. Results

A total of 107 patients met the criteria for inclusion in the study. Patient demographics and main findings are summarised in Table 1. The mean age was 50.9 years and 98 patients (92%) were male.

**Figure 1:**
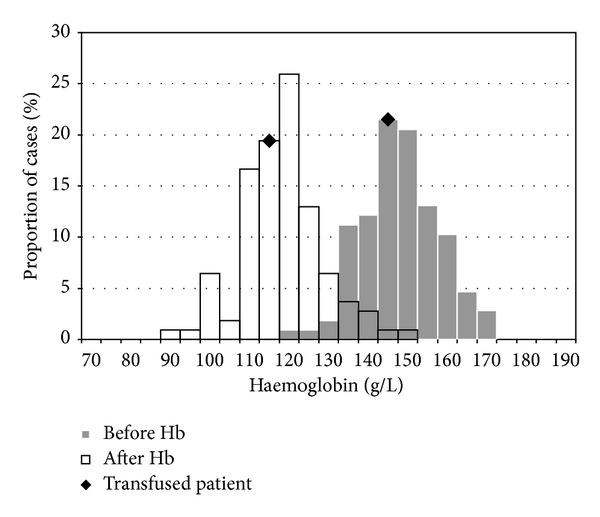
Pre- and postoperative haemoglobin measurements.

**Figure 2:**
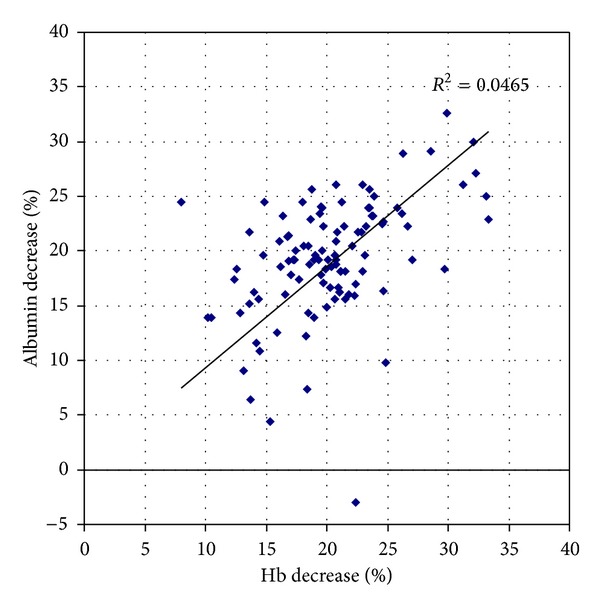
Comparison of percentage haemoglobin and albumin decrease after surgery.

The mean preoperative haemoglobin was 148.1 g/L (range: 121 g/L–171 g/L). The mean postoperative haemoglobin was 118.1 g/L (range: 92 g/L–149 g/L) (Figure 1). The mean decrease in haemoglobin observed between blood tests was 30.1 g/L. The percentage decrease in haemoglobin correlated statistically with the decrease in albumin levels observed on postoperative blood testing (Pearson's rho = 0.216, *P* = 0.012; Figure 2).

Of the 107 patients who underwent hip resurfacing, there was one patient who required blood transfusion. This patient was a 53-year-old male with multiple medical comorbidities including obesity (160 kgs, body mass index of 46.7 kg/m^2^) and obstructive sleep apnoea using continuous positive airway pressure (CPAP) machine and was previously a heavy smoker. He recorded a modest reduction in haemoglobin in association with his surgery in comparison to the remainder of the cohort (preoperative 147 g/L, postoperative 118 g/L); however, on the basis of persistent fatigue and dyspnoea with presyncopal episodes a blood transfusion of packed red cells was undertaken. The transfusion significantly improved his symptoms and rehabilitation outcome.

The cohort mean length of stay was 3.5 days (median duration: 3 days). No statistical correlation was observed between haemoglobin levels and length of stay (*P* > 0.2).

There were no recorded deaths, infections, or revisions of implants within the time period.

## 4. Discussion

The rational use of allogeneic blood products in patients undertaking major surgical intervention is assisted by the use of protocols and criteria to guide treating physicians [10, 11]. This data, however, questions the validity of routine testing of haemoglobin levels in the perioperative period to guide blood management after resurfacing hip arthroplasty, an intervention which is typically undertaken in a younger population with less medical comorbidity.

The overall transfusion rate in this series is under 1% (0.9%, 95% confidence interval: 0.1%–5.8%). The sole patient requiring blood transfusion, who was somewhat atypical for a patient undertaking resurfacing arthroplasty in the context of having multiple medical comorbidities, had a reasonable postoperative haemoglobin level at 118 g/L. The decision to proceed with blood transfusion was made on clinical grounds of symptomatic anaemia despite the reasonable postoperative haemoglobin level recorded. In no other patient was significant clinical anaemia detected that precluded early mobilisation or relatively early hospital discharge. It should be noted that intraoperative or postoperative tranexamic acid was used in no patient within this study, although the authors acknowledge that the wide volume infiltration of local anaesthetic including adrenaline used may have reduced intraoperative blood loss.

Adding doubt to the relevance of early postoperative haemoglobin testing was the observed strong correlation with albumin levels in the postoperative period. This finding suggests the postoperative reduction in haemoglobin levels observed is representative of haemodilution rather than blood loss during surgery [12, 13]. While routine blood testing on day 2 after surgery may reduce the influence of intraoperative crystalloid administration on the postoperative haemoglobin results obtained, the absence of requirement to perform blood transfusion on clinical grounds in all but 1 patient in this series remains consistent.

We recommend from this data that routine testing of haemoglobin in the perioperative period associated with hip resurfacing arthroplasty is seldom necessary. In particular, this applies to postoperative blood testing on day one after intervention, as the results are potentially poorly representative of absolute blood loss. Reduction in unnecessary blood testing in the perioperative period reduces overall health care costs associated with the intervention, simplifies nursing care, and reduces patient discomfort. The authors do, however, recommend that postoperative blood testing can be of value in patients who demonstrate persistent symptomatic anaemia in whom blood transfusion is considered on clinical grounds. We also recommend that preoperative blood testing may be of value to detect and evaluate preoperative anaemia, but only when performed substantially in advance of the intervention to allow correction prior to surgery.

While this study has only evaluated patients undertaking hip resurfacing arthroplasty, who tend to be younger with less medical comorbidity and greater physiologic reserve to withstand moderate postoperative anaemia, we believe the results are also applicable to suitable patients undertaking conventional total hip replacement procedures, particularly as the soft tissue releases and degree of potential blood loss are typically less in total hip arthroplasty.

Limitations of the study include the observational nature of the data and the fact that the results have been drawn from the outcomes of a single surgeon experienced in resurfacing arthroplasty operating in a single institution. Accordingly, these outcomes may not be reflective of general orthopaedic practice [14]. In addition, formal measurement of intraoperative blood loss was not undertaken.

## 5. Conclusion

In summary, these data do not support the routine testing of haemoglobin associated with hip resurfacing arthroplasty.

## Figures and Tables

**Table 1:** Summary of patient demographics and findings. Unless otherwise stated, values shown are mean (range).

Number of patients	107
Number of males (*n*; %)	98 (92%)
Age	50.9 yrs (28.7–66.2 yrs)
Preoperative haemoglobin	148.2 g/L (121–171 g/L)
Postoperative haemoglobin	118.1 g/L (92–149 g/L)
Hb drop	30.1 g/L (12–53 g/L)
Percentage Hb drop	20.3% (8.0–33.3%)
Preoperative albumin	45.6 g/L (34–52 g/L)
Postoperative albumin	36.7 g/L (32–44 g/L)
Albumin drop	8.9 g/L (−1–16 g/L)
Percent albumin drop	19.3% (−2.9–32.7%)
Patients requiring blood transfusion	1
Length of stay	3.5 days (1–7 days)
